# Editorial: Community series in the role of monocytes/macrophages in autoimmunity and autoinflammation, volume II

**DOI:** 10.3389/fimmu.2025.1699650

**Published:** 2025-09-16

**Authors:** Naoki Iwamoto, Atsushi Kawakami, Sachiko Miyake, Keishi Fujio

**Affiliations:** ^1^ Department of Immunology and Rheumatology, Division of Advanced Preventive Medical Sciences, Nagasaki University Graduate School of Biomedical Sciences, Nagasaki, Japan; ^2^ Department of Immunology, Juntendo University Faculty of Medicine, Tokyo, Japan; ^3^ Department of Allergy and Rheumatology, Graduate School of Medicine, The University of Tokyo, Tokyo, Japan

**Keywords:** monocyte, macrophage, autoimmune disease, M1 macrophage, M2 macrophage, interferon

Monocytes and macrophages are central innate immune cells that regulate inflammatory responses, tissue remodeling, and host defense (1). Their dysregulation contributes to a wide range of autoimmune and inflammatory diseases through overproduction of cytokines and chemokines, aberrant activation, and maladaptive repair (2, 3). In recent years, new investigative techniques such as single-cell technologies have highlighted the remarkable heterogeneity of these cells, moving the field beyond oversimplified classifications toward recognition of diverse and context-dependent states. For example, a recent single-cell study in systemic sclerosis (SSc) identified EGR1^+^ CD14^+^ monocytes associated with scleroderma renal crisis and tissue-damaging macrophages, underscoring how cellular diversity contributes to organ-specific pathology (4). This recognition has spurred renewed interest in their role as both drivers of pathology and potential therapeutic targets. While the previous editorial in 2022 (topic title: the role of monocytes/macrophages in autoimmunity and autoinflammation) focused broadly on macrophage polarization, trained immunity, and osteoclastogenesis across various autoimmune diseases such as rheumatoid arthritis, systemic lupus erythematosus (SLE), and vasculitis (5), the present Research Topic serves as a continuation, featuring new original studies and reviews that specifically highlight the evolving relationship between monocytes, macrophages, and autoimmune diseases.

Hyperactivation and excessive infiltration of macrophages in tissue are hallmarks of hemophagocytic lymphohistiocytosis (HLH), Hu et al. investigated the treatment of HLH with chidamide, a histone deacetylase inhibitor, in combination with etoposide and glucocorticoids. They reported high response rates and favorable safety, indicating that epigenetic modulation can restrain macrophage overactivation and may offer new options for cytokine storm syndromes. Hovd et al. examined murine models of Sjögren’s disease(SjD) and identified a subset of podoplanin+CX3CR1+ macrophages enriched in inflamed salivary glands. These cells localized in the parenchyma during the acute inflammatory phase and near tertiary lymphoid structures (TLSs) in the later stages of inflammation, and appeared to promote lymphoid neogenesis, suggesting that macrophages play a dual role by contributing to both pro-inflammatory and anti-inflammatory processes. In complementary patient-based studies, Yan et al. conducted DNA microarray analysis followed by single-cell RNA sequencing of salivary gland tissue from SjD patients, and demonstrated that Toll-like receptor (TLR)8+ macrophages infiltrate the glands, express co-stimulatory molecules such as CD86, and activate T cells. Together, these findings emphasize that macrophages orchestrate both tissue architecture and adaptive immune responses in glandular autoimmunity.

In SSc-associated interstitial lung disease (ILD), Gotelli et al. described a distinct population of circulating (peripheral blood) and alveolar fluid hybrid TLR4+M2 marker (CD163, CD204 and CD206) positive cells. These cells simultaneously expressed fibrotic and inflammatory markers, suggesting dual pathogenic potential, and their presence in blood offers a potential biomarker for disease progression. Kuga et al. reviewed evidence that, although plasmacytoid dendritic cells have long been recognized as the canonical source of type I interferon(IFN)s in SLE, monocytes—particularly those with senescence-like features—also produce substantial amounts of these cytokines, highlighting an intriguing additional pathway of IFN generation in the disease. They highlighted cGAS–STING and TLR pathways activated by self-nucleic acids and neutrophil extracellular traps, broadening the scope of IFN biology and suggesting that, in addition to existing therapies such as anifrolumab that target the IFN axis, strategies directed specifically at monocyte-driven IFN production may represent a promising future approach.

Izuka et al. provided a comprehensive overview of monocytes and macrophages in idiopathic inflammatory myopathies (IIMs), including dermatomyositis and immune-mediated necrotizing myopathy. They noted that macrophages infiltrate skeletal muscle and contribute to tissue injury through pro-inflammatory mediators and reactive oxygen species, but also participate in regeneration and repair through specialized subsets. In the muscle, these macrophages are involved in phagocytosis and tissue damage, whereas in the lung they are associated with ILD, often accompanied by elevated ferritin and type I IFN responses. Advances in single-cell transcriptomics have revealed heterogeneity that may serve as biomarkers of prognosis and guide reprogramming therapies, and potential therapeutic approaches include targeting chemokine receptors, cytokine pathways, oxidative stress responses, or IFN signaling. Finally, Wang et al. reviewed the role of macrophages in rosacea, a chronic inflammatory skin disease characterized by flushing and persistent erythema. They described how macrophages regulate angiogenesis, oxidative stress, and fibrosis, underscoring their plasticity and potential as therapeutic targets even in dermatological conditions not traditionally linked to systemic autoimmunity.

Collectively, these contributions reveal several unifying themes. Macrophages *in vivo* are highly plastic and exist in hybrid states shaped by tissue-specific cues, as exemplified by TLR4+M2 cells in SSc-ILD or podoplanin+ subsets in SjD. They serve as critical bridges between innate and adaptive immunity, both sensing nucleic acids via TLRs and cGAS–STING and delivering co-stimulatory signals to T cells. Their functions are profoundly organ-specific, acting as fibrosis drivers in lung, lymphoid architects in salivary glands, IFN producers in lupus, tissue repair facilitators in myositis, and vascular remodelers in rosacea. Importantly, translational relevance is increasingly evident: chidamide in HLH demonstrates therapeutic modulation of macrophages in hyperinflammation, anifrolumab exemplifies IFN pathway targeting in lupus, and emerging strategies aim at selective macrophage reprogramming in muscle and skin diseases.

In conclusion, monocytes and macrophages emerge from these studies not as static entities but as dynamic populations integrating signals from their microenvironment and determining the trajectory of immune-mediated tissue damage or repair. We are pleased that this issue dedicated to “*The role of monocytes/macrophages in autoimmunity and autoinflammation, volume II*” has gathered a range of innovative studies and reviews, advancing our understanding of the diverse functions of monocytes and macrophages and their relevance to autoimmune and autoinflammatory diseases.

## References

[1] Ginhoux F Jung S. Monocytes and macrophages: developmental pathways and tissue homeostasis. Nat Rev Immunol. (2014) 14:392–404. doi: 10.1038/nri3671, PMID: 24854589

[2] Park MD Silvin A Ginhoux F Merad M. Macrophages in health and disease. Cell. (2022) 185:4259–79. doi: 10.1016/j.cell.2022.10.007, PMID: 36368305 PMC9908006

[3] Italiani P Boraschi D. From monocytes to M1/M2 macrophages: phenotypical vs. Functional differentiation. Front Immunol. (2014) 5:514. doi: 10.3389/fimmu.2014.00514, PMID: 25368618 PMC4201108

[4] Shimagami H Nishimura K Matsushita H Metsugi S Kato Y Kawasaki T. Single-cell analysis reveals immune cell abnormalities underlying the clinical heterogeneity of patients with systemic sclerosis. Nat Commun. (2025) 16:4949. doi: 10.1038/s41467-025-60034-7, PMID: 40527894 PMC12174364

[5] Kawakami A Iwamoto N Fujio K. Editorial: The role of monocytes/macrophages in autoimmunity and autoinflammation. Front Immunol. (2022) 13:1093430. doi: 10.3389/fimmu.2022.1093430, PMID: 36483549 PMC9723365

